# Impact of multiple long term conditions on hospital admission and mortality during winter: importance of linked, population scale healthcare data

**DOI:** 10.1136/bmjmed-2024-001114

**Published:** 2024-11-12

**Authors:** Jonathan Adam Batty, Lesley Smith, Marlous Hall

**Affiliations:** 1 Leeds Institute of Cardiovascular and Metabolic Medicine, University of Leeds, Leeds, UK; 2 Leeds Institute for Data Analytics, University of Leeds, Leeds LS2 9NL, UK; 3 Leeds Institute of Clinical Trials Research, University of Leeds, Leeds, UK

**Keywords:** Epidemiology, Public health, Health policy

The availability of population scale data as a result of the covid-19 pandemic has provided actionable insights for policy makers but needs to expand beyond pandemic planning, argue Batty and colleagues.

Winter poses a substantial challenge to healthcare systems as a consequence of colder weather exacerbating pre-existing health issues, increased circulation of seasonal viruses, social factors (increased isolation and loneliness), and difficulties at the systems level (higher bed occupancy and greater staff absences).^1 2^ The growing prevalence of multimorbidity—defined as the presence of multiple long term conditions in an individual^3^—further exacerbates these pressures.

Although the impact of multimorbidity on hospital admission and all cause mortality is well documented,^4^ the effect of specific combinations of long term conditions in the winter remains unclear. To investigate this, Islam and colleagues (doi:10.1136/bmjmed-2024-001016) conducted a population based, retrospective cohort study with coverage of 48.2 million adults in England, using routinely collected, linked primary and secondary care data.^5^ The authors ascertained 59 long term conditions and reduced these to 19 groups based on clinician, policymaker, and patient input. The overall prevalence of multimorbidity was 31.2%, in line with previous estimates in this population.^6^ Individuals with cancer, kidney disease, cardiovascular disease, and type 2 diabetes mellitus were observed to be 11-fold (95% confidence interval (CI) 9.4 to 12.7) more likely to be admitted to hospital during winter (December 2021 to March 2022) than those without long term conditions. Those individuals with kidney disease, cardiovascular disease, dementia, and osteoarthritis were 24.3-fold (95% CI 19.1 to 30.4) more likely to die during winter than those without long term conditions.

Multimorbidity could increase hospital admission and mortality during winter by increasing vulnerability to the development of critical illness in response to seasonal viruses as a result of impaired control of viral replication, dysregulated host immune responses, and diminished physiological reserves.^7^ Coexisting frailty and disability could increase the risk of unexpected social care needs that cannot be met in the community during winter.^8^ Specific long term conditions might also mediate excess adverse outcomes (eg, resting blood pressure peaks in winter, increasing the risk of cardiovascular events).^9^

This study by Islam and colleagues showed that individuals with specific combinations of long term conditions were at greater risk of hospital admission and mortality during winter after accounting for age, sex, ethnic group, and socioeconomic deprivation, which has several potential key implications for health policy. Firstly, the large number of combinations of long term conditions observed in this study reinforces the need for joined-up, patient centred models of care; many long term conditions continue to be managed by hospital based specialists, with the overall care of the patient coordinated in primary care. New models of integrated community, hospital, and social care are likely to offer better support to those people living with multimorbidity, but will require paradigm shifts in commissioning and organisational culture. Secondly, the most adverse combinations included multiple long term conditions with common risk factors, including smoking, obesity, and a sedentary lifestyle, suggesting that prioritising the management of these risk factors earlier in an individual's life course might have synergistic benefits in preventing multimorbidity and consequent adverse clinical outcomes.

The future development of this work might help identify those people at greatest risk of unplanned admission and enable targeted, evidence based interventions to prevent hospital admission,^10^ including enhanced community based care^11^ and vaccination against winter viruses.^12^ However, Islam and colleagues recognise that this descriptive, observational study was able to estimate the population level burden of hospital admission and mortality, rather than derive a clinical prediction model at the individual level (a pre-requisite for clinical implementation). The authors also assert that further work is required to understand whether the associations identified between combinations of long term conditions, hospital admission, and mortality have any causal interpretation, and that their selection of long term conditions is key to their findings (ie, alternative selection of conditions could lead to different findings). Therefore, further work is still required for data such as these to directly inform clinical practice and to allow robust translation of these findings into risk stratification for individuals.

This study had several methodological strengths. Firstly, the use of individual level primary and secondary care data, linked at whole population scale, enabled the robust and representative inference required to inform healthcare policy. Access to these high quality data were made possible by the CVD-COVID-UK/COVID-IMPACT Consortium and the combined work of Health Data Research UK and the British Heart Foundation Data Science Centre, with secure access provided through trusted research environments.^13^ These data became available as a result of changes to legislation made during the covid-19 pandemic to enable timely, data driven, policy responses, but data linkage at this scale remains restricted to research focused on pandemic planning.^14^ Secondly, the authors evaluated specific combinations of long term conditions rather than limiting their analysis to the binary definition of multimorbidity. This approach has enabled specific, actionable insights, including finding that certain two-condition dyads (eg, cardiovascular disease and dementia) were associated with a greater risk of hospital admission and death than many combinations of three, four, and five diseases. Future research on multiple long term conditions should continue to evaluate which conditions individuals have,^15^ in addition to incorporating metadata regarding order of accrual,^16^ duration, and severity.

This study also highlights the considerable methodological complexity of studying the synergistic impact of many long term conditions. There are 5.8×10^17^ hypothetical combinations of 59 conditions, of which over 500 000 were observed by the authors. Enumerating and meaningfully interpreting combination-outcome associations at this scale remains intractable, even in a whole population study. Accordingly, a practical decision was made to simplify the initial list of conditions to the 19 most important conditions, and to report only the top associations by effect size. In future, sophisticated methods including clustering,^17^ regularisation^18^ and dimensionality reduction^19^ may reduce complexity while preserving key interactions between a larger number of long term conditions. Finally, the authors identified combinations of long term conditions most associated with adverse outcomes during winter, but their study design was not able to quantify the excess risk beyond that observed in non-winter months, or comment on the preventability of hospital admissions and deaths.

In conclusion, this study demonstrated that multimorbidity patterns are a major determinant of hospital admission and mortality during winter. In the broader context of winter pressures and increasing multimorbidity, it underscores the need for methods that can identify individuals at high risk of preventable hospital admission and mortality, and strategies to mitigate the risk observed for those people with the most adverse combinations of long term conditions. Lastly, although other examples of high impact analyses of population scale data are emerging,^20 21^ it is of critical importance to make these data available beyond the context of pandemic planning, in order to answer some of the most important questions in healthcare, health policy, and society.
